# Impact of Tyrosine Kinase Inhibitors on Thyroid Function in Chronic Myeloid Leukemia: A Systematic Review

**DOI:** 10.7759/cureus.85196

**Published:** 2025-06-01

**Authors:** Nana Sardarova, Tirath Patel, Tahani M Abugoukh, Daniel Kim, Samad Yousuf, Mohammed Hammoude

**Affiliations:** 1 Internal Medicine, Henry Ford Health System, Warren, USA; 2 Health Science, American University of Antigua, Saint John's, ATG; 3 Medicine, Shendi University, Cedar Rapids, USA; 4 Medicine, Kansas City University, Kansas City, USA; 5 Endocrinology, Henry Ford Health System, Detroit, USA

**Keywords:** autoimmune thyroiditis, chronic myeloid leukemia, cml, hyperthyroidism, hypothyroidism, thyroid disease, tki, tyrosine kinase inhibitor

## Abstract

Tyrosine kinase inhibitors (TKIs) have significantly improved outcomes in chronic myeloid leukemia (CML), shifting it from a fatal to a manageable chronic condition. Despite their clinical benefits, TKIs have been increasingly associated with endocrine-related adverse effects, most notably thyroid dysfunction. This systematic review explores the prevalence, clinical features, underlying mechanisms, and prognostic implications of TKI-induced thyroid abnormalities in CML patients. Following Preferred Reporting Items for Systematic Reviews and Meta-Analyses (PRISMA) 2020 guidelines, a comprehensive literature search was conducted across four databases, namely, PubMed, Excerpta Medica database (Embase), ClinicalKey, and Google Scholar, yielding 4,803 records. After applying inclusion and exclusion criteria and quality assessments using the Newcastle-Ottawa Scale (NOS) and A Measurement Tool to Assess Systematic Reviews (AMSTAR 2), eight studies (six observational studies and two systematic reviews) were included in the final analysis. Subclinical hypothyroidism emerged as the most frequently reported thyroid dysfunction, particularly associated with imatinib and second-generation TKIs such as nilotinib and dasatinib. Proposed mechanisms include destructive thyroiditis, reduced iodide uptake, regression of thyroid vasculature via vascular endothelial growth factor (VEGF) inhibition, inhibition of monocarboxylate transporter 8 (MCT8)-mediated thyroid hormone transport, and increased deiodinase activity leading to hormone inactivation. Notably, several studies identified an association between autoimmune thyroiditis and improved molecular response to TKIs, suggesting a potential role for thyroid autoimmunity as a biomarker of therapeutic efficacy. While most thyroid abnormalities were subclinical and did not necessitate treatment, overt hypothyroidism required thyroid hormone replacement and endocrine follow-up. This review emphasizes the importance of routine thyroid function monitoring during TKI treatment and highlights the potential prognostic implications of thyroid autoimmunity. Future large-scale, prospective studies are needed to establish standardized monitoring protocols and clarify the clinical significance of thyroid changes in optimizing CML management.

## Introduction and background

Chronic myeloid leukemia (CML) is a hematopoietic stem cell disorder characterized by the malignant transformation of a pluripotent stem cell. The disease is driven by the presence of the Philadelphia chromosome (Ph), a genetic abnormality formed through a reciprocal translocation between chromosomes 9 and 22, noted as t(9;22)(q34;q11). This rearrangement creates the BCR-ABL1 fusion gene, which produces a constitutively active protein tyrosine kinase that leads to uncontrolled cell growth [1]. CML has undergone a remarkable transformation in its management over the past two decades, largely due to the advent of tyrosine kinase inhibitors (TKIs). TKIs are targeted molecules structurally similar to ATP molecules, designed to competitively bind to the ATP-binding site of the BCR-ABL1 tyrosine kinase. This competitive inhibition prevents phosphorylation by TK, consequently disrupting the TK-driven oncogenic signaling pathways [2]. Imatinib became the first TKI to receive approval from the US Food and Drug Administration (FDA) in 2001 for the treatment of CML [3]. Currently, six BCR:ABL1 TKIs are approved by the US FDA, including five (imatinib, dasatinib, bosutinib, nilotinib, and asciminib) approved for first-line therapy, and five (dasatinib, bosutinib, nilotinib, ponatinib, and asciminib) approved for use in cases with disease progression following initial treatment [4].

TKIs have shifted the prognosis of CML from a once-fatal illness to a manageable chronic condition with near-normal life expectancy for many patients. As TKIs became more widely used, various side effects were observed, including thyroid dysfunction [5]. The thyroid gland plays a critical systemic role in regulating metabolism, cardiovascular function, thermoregulation, and neurocognitive health through the secretion of thyroid hormones. Several possible mechanisms have been suggested to explain thyroid dysfunction, such as direct toxic effects of TKIs on follicular cells, triggering destructive thyroiditis, accelerated thyroid hormone clearance, regression of thyroid capillaries caused by inhibition of vascular endothelial growth factor (VEGF), and reduced iodide uptake [6]. The adverse effects can present in various forms, including hypothyroidism, hyperthyroidism, or thyroiditis, and may significantly affect patients' quality of life and present clinical challenges during long-term treatment. Additionally, interest in TKI-induced hypothyroidism has grown following reports suggesting that certain side effects, such as hypertension, hypothyroidism, and hand-foot syndrome, might act as potential predictive biomarkers of treatment efficacy [7]. Therefore, understanding and addressing TKI-induced thyroid abnormalities have become increasingly important in the comprehensive care of patients with CML. This review article aims to explore the thyroid-related adverse effects of TKI therapy in CML patients to provide guidance for clinicians on monitoring and managing these effects effectively.

## Review

Methods

Protocol and Registration

This systematic review was designed using the Preferred Reporting Items for Systematic Review and Meta-analyses (PRISMA) 2020 guidelines [8]. It was registered in the International Prospective Register of Systematic Reviews (PROSPERO) under the registration number CRD420250627959. The protocol is available at https://www.crd.york.ac.uk/PROSPERO/view/CRD420250627959.

Search Strategy

A systematic review was conducted by using the following electronic databases: PubMed, Excerpta Medica database (Embase), ClinicalKey, and Google Scholar. Eligible articles were thoroughly explored and identified by a search on 30^th^ November 2024. Medical Subject Heading (MeSH) search strategy with a Boolean approach was employed using the following concepts: ("leukemia, myelogenous, chronic, bcr abl positive/blood"[MeSH Terms] OR "leukemia, myelogenous, chronic, bcr abl positive/complications"[MeSH Terms] OR "leukemia, myelogenous, chronic, bcr abl positive/drug therapy"[MeSH Terms]) AND ("thyroid diseases"[MeSH Terms] OR "thyroid disease"[Text Word]). Additional articles were identified using the regular keyword "TKI, thyroid" for search in PubMed. Further details of the keywords applied for each database search strategy are described in Table 1. Duplicate articles were manually removed from PubMed and Embase using EndNote (Clarivate, London, UK). Subsequently, two authors independently screened 1,413 reports based on titles and abstracts to assess their relevance. The remaining articles were evaluated for full-text availability, and inclusion and exclusion criteria were applied for further filtration and eligibility assessment. Data extraction was performed independently by two reviewers using a standardized form, with discrepancies resolved by a third reviewer. 

**Table 1:** Details of the search strategy Embase: Excerpta Medica database

Database	Search strategy	Papers identified
PubMed MeSH terms	("leukemia, myelogenous, chronic, bcr abl positive/blood"[MeSH Terms] OR "leukemia, myelogenous, chronic, bcr abl positive/complications"[MeSH Terms] OR "leukemia, myelogenous, chronic, bcr abl positive/drug therapy"[MeSH Terms]) AND ("thyroid diseases"[MeSH Terms] OR "thyroid disease"[Text Word])	24
PubMed Keywords	TKI Thyroid	345
Embase	'chronic myeloid leukemia' AND TKI AND 'thyroid disease'	25
ClinicalKey	CML, Tyrosine Kinase Inhibitor, Thyroid	509
Google Scholar	Thyroid Dysfunction in CML Patients on TKI Therapy	3,900
Total		4,803

Eligibility Criteria

The review included studies meeting the following criteria: adult and pediatric patients diagnosed with CML as the study population; interventions involving treatment with TKIs; reported outcomes related to thyroid effects, including hypothyroidism, hyperthyroidism, autoimmune thyroiditis, thyroid function changes, or mechanistic insights into TKI-induced thyroid dysfunction; study designs encompassing clinical trials (randomized or non-randomized), case-control, cross-sectional, cohort studies, and systematic reviews and meta-analyses; publication in English or availability of an English translation; and publication dates from 2010 onward.

Articles were excluded from the review based on the following criteria: studies that did not involve patients with CML or exclusively focused on other types of cancers; studies investigating thyroid dysfunction without specific reference to TKI therapy or those involving non-TKI medications; articles that lacked thyroid-related outcomes, such as those reporting only generic quality of life measures without thyroid assessment; study designs limited to literature reviews, case reports, case series, editorials, commentaries, opinion pieces, or animal studies; articles not published in English or without an available English translation; and studies published before 2010.

Quality Appraisal Tools

**Figure 1:**
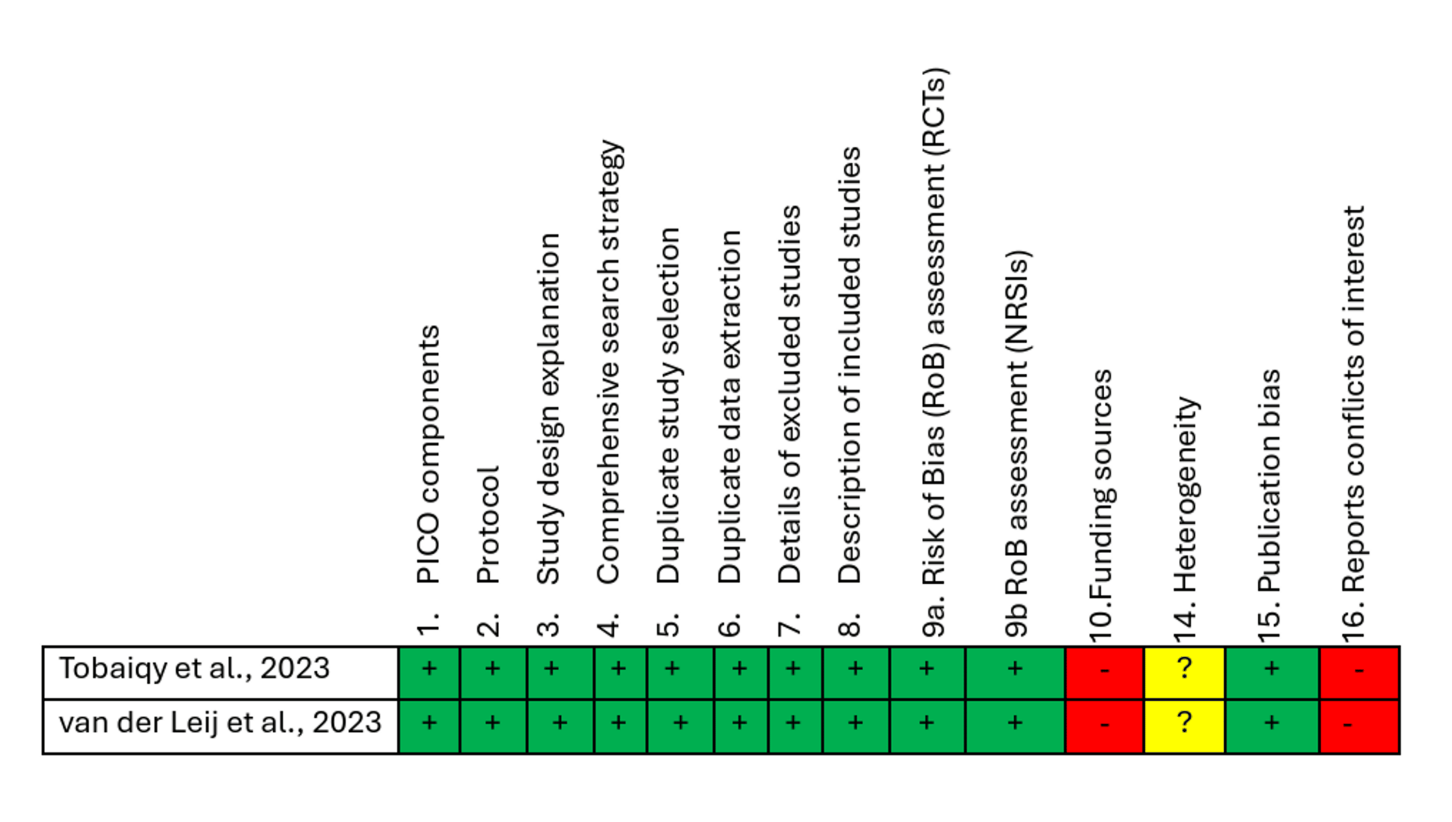
A Measurement Tool to Assess Systematic Reviews (AMSTAR 2) Systematic reviews that underwent quality appraisal: Tobaiqy et al., 2023 [16], van der Leij et al., 2023 [17]. PICO: Patient, Intervention, Comparison, and Outcome; RCTs: randomized controlled trial; NRSIs: Non-randomized Studies of Interventions

After applying the inclusion and exclusion criteria, the list was narrowed down to nine reports, and quality appraisal tools were applied to each type of study. Three authors assessed the quality of included studies and the risk of bias using the Newcastle-Ottawa Scale (NOS) for observational studies (Table 2) and the A Measurement Tool to Assess Systematic Reviews (AMSTAR 2) for systematic reviews (Figure 1). The reports that scored 70% or higher were selected for the final systematic review.

**Table 2:** Newcastle-Ottawa quality assessment scale cohort studies/cross-sectional studies and case-control studies

Study	Selection	Comparability	Outcome/Exposure	Risk of bias
Rodia et al., 2021 [9]	***	**	***	Low risk of bias
Khaleel et al., 2018 [10]	****	*	**	Low risk of bias
Chawalitmongkol et al., 2023 [11]	***	*	***	Low risk of bias
Ahmed et al., 2024 [12]	****	*	***	Low risk of bias
Kim et al., 2010 [13]	***	*	***	Low risk of bias
Luna et al., 2024 [14]	***	*	***	Low risk of bias
Allahyari et al, 2016 [15]	**	*	***	Moderate risk of bias

Results

**Figure 2:**
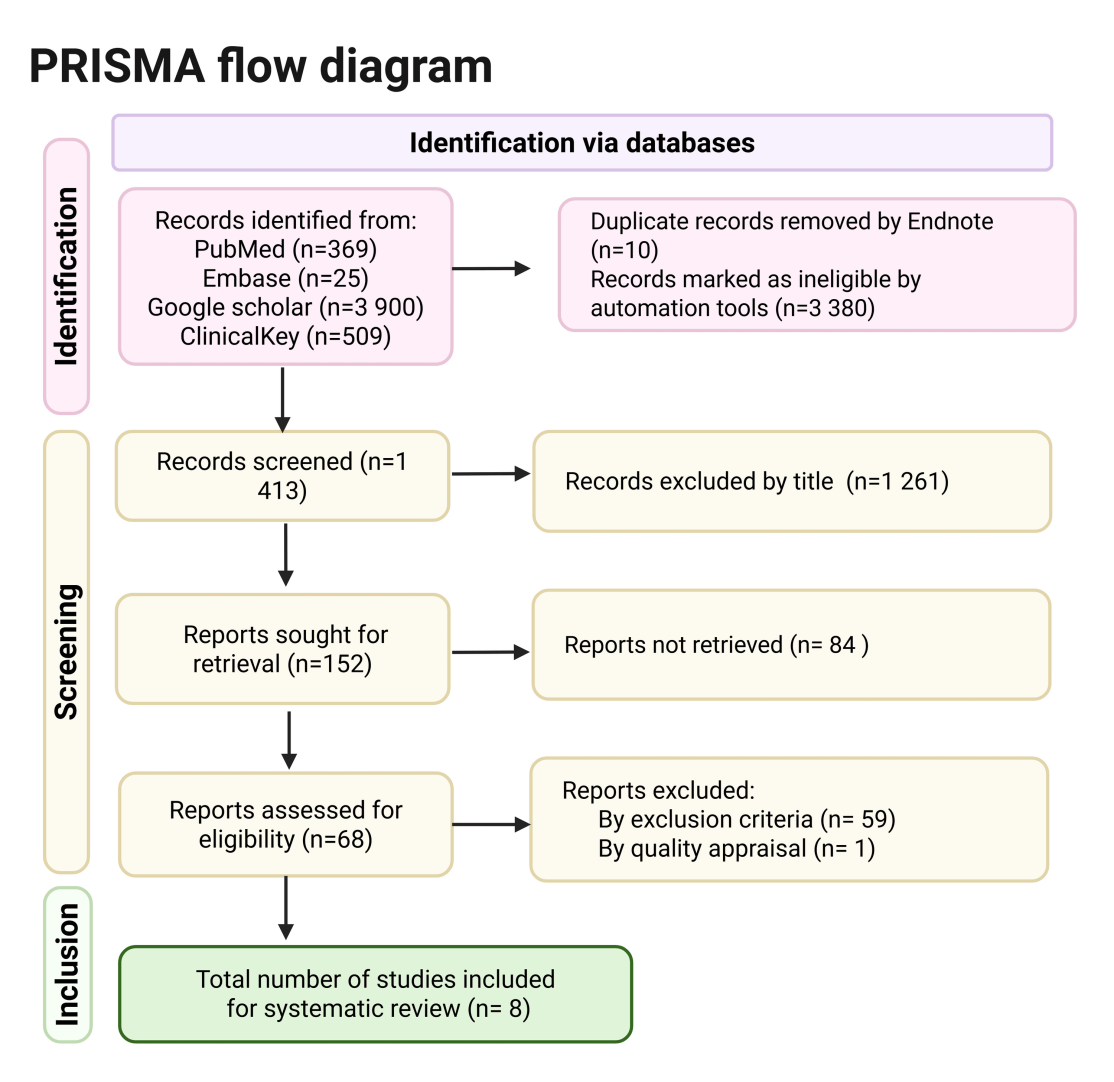
Preferred Reporting Items for Systematic Review and Meta-analyses (PRISMA) 2020 Embase: Excerpta Medica database

Out of nine articles that underwent quality assessment, eight fulfilled the required standards and were subsequently included in the review. The final selection incorporated six observational studies and two systematic reviews. The search results are presented in Figure 2 as a Preferred Reporting Items for Systematic Reviews and Meta-Analyses (PRISMA) flow diagram.

Data Synthesis

Due to substantial clinical and methodological heterogeneity across the included studies, such as differences in TKI types, study designs, populations, diagnostic criteria for thyroid dysfunction, and inconsistent reporting of effect estimates, a quantitative synthesis (meta-analysis) was not deemed appropriate. Therefore, no effect sizes were pooled, and no statistical model was applied. Instead, we conducted a narrative synthesis of the findings, as recommended in PRISMA 2020 for systematic reviews where quantitative synthesis is not feasible [8]. Study results are summarized descriptively, with proportions and confidence intervals reported when available. No statistical software or meta-analytic packages were used.

Study Characteristics

The characteristics of the eight included studies are shown in Table 3.

**Table 3:** Summary of studies TKIs: tyrosine kinase inhibitors; CML: chronic myeloid leukemia

Author and year of publication	Title	Country	Type of Research	Number of subjects	Outcome
Rodia et al.,2021 [9]	Thyroid autoimmunity and hypothyroidism are associated with deep molecular response in patients with chronic myeloid leukemia on tyrosine kinase inhibitors.	Italy	Retrospective cohort study	69	34.8% of patients had thyroid abnormalities, 30.4% showed thyroid autoimmunity, clinical and subclinical hypothyroidism were found in 5.8%, and subclinical hyperthyroidism in 4.3%; second-generation TKIs were more associated with Hashimoto’s thyroiditis (43.7% vs. 18.9%, p = 0.03), and the deep molecular response was significantly linked to euthyroid (53.8%) and hypothyroid Hashimoto’s (15.4%) compared to major molecular response (7% and 0%, p ≤ 0.02).
Khaleel et al., 2018 [10]	Thyroid dysfunction in chronic myeloid leukemia patients on nilotinib	Iraq	Cross‑sectional study	33	About 10% of patients had hypothyroidism, 3% were hyperthyroid, and the remaining 87% had normal thyroid function. Thyroid-stimulating hormone levels were significantly higher in the study group compared to the control group (P < 0.05).
Chawalitmongkol et al., 2023 [11]	Prevalence and Associated Factors for Thyroid Dysfunction Among Patients on Targeted Therapy for Cancers: A Single-Center Study from Thailand	Thailand	Cross-sectional study	68	Thyroid dysfunction was observed in 14.7% of patients, with the majority experiencing subclinical hypothyroidism. The most frequently used TKIs among those with thyroid dysfunction were imatinib (10.8%) and sunitinib (100%).
Ahmed et al., 2024 [12]	Subclinical Hypothyroidism Following Imatinib Use in Nigerian BCR: ABL1-positive Chronic Myeloid Leukemia Patients: A Prospective Cohort Study	Nigeria	Prospective cohort study	50	Subclinical hypothyroidism was identified in 14% of the patients with CML at six months on imatinib.
Kim et al., 2010 [13]	Thyroid Dysfunction Caused by Second-Generation Tyrosine Kinase Inhibitors in Philadelphia Chromosome-Positive Chronic Myeloid Leukemia	Germany	Prospective cohort study	73	45% of participants exhibited at least one abnormality in their thyroid function tests. 7% of patients under treatment with nilotinib had evidence of autoimmune thyroiditis with an episode of hyperthyroidism preceding hypothyroidism.
Luna et al., 2024 [14]	Clinical Profile of Non-thyroidal Cancer Patients with Tyrosine Kinase Inhibitor-induced Thyroid Dysfunction in the University of Santo Tomas Hospital, Philippines: A 5-Year Single-center Retrospective Study	Philippines	Retrospective cohort study	60	85% of CML patients (14 people) in this study had euthyroid state. Two out of 14 CML patients developed hyperthyroidism.
Tobaiqy et al., 2023 [16]	The prevalence of hepatic and thyroid toxicity associated with imatinib treatment of chronic myeloid leukemia: a systematic review	Unclear	Systematic review	167	14 cases of thyroid dysfunction (8.4%, n = 14/167) were reported as adverse events associated with imatinib use in patients with CML. These included one instance of severe autoimmune thyroiditis, two cases of mild hypothyroidism and hyperthyroidism, three cases of goiter with varying severity, and eight cases involving other forms of thyroid abnormalities.
Van der Leij., 2023 [17]	Thyroid dysfunction during treatment with systemic antineoplastic therapy for childhood cancer	Netherlands	Systematic review	38	Only two patients were identified with subclinical hypothyroidism and were administered thyroid hormone therapy.

Discussion

In recent years, TKIs have become an important class of anti-cancer drugs, showing effective results in treating various types of carcinomas. Although typically viewed as having lower toxicity compared to traditional cytotoxic chemotherapy, they have different side effects depending on which molecules they target. Thyroid dysfunction related to TKI therapy is now recognized as a frequent adverse effect associated with certain TKIs [18]. Our review data are centered on patients with CML during treatment with first- and second-generation TKIs in relation to thyroid outcomes.

Mechanisms of TKI-induced Thyroid Dysfunction

**Figure 3:**
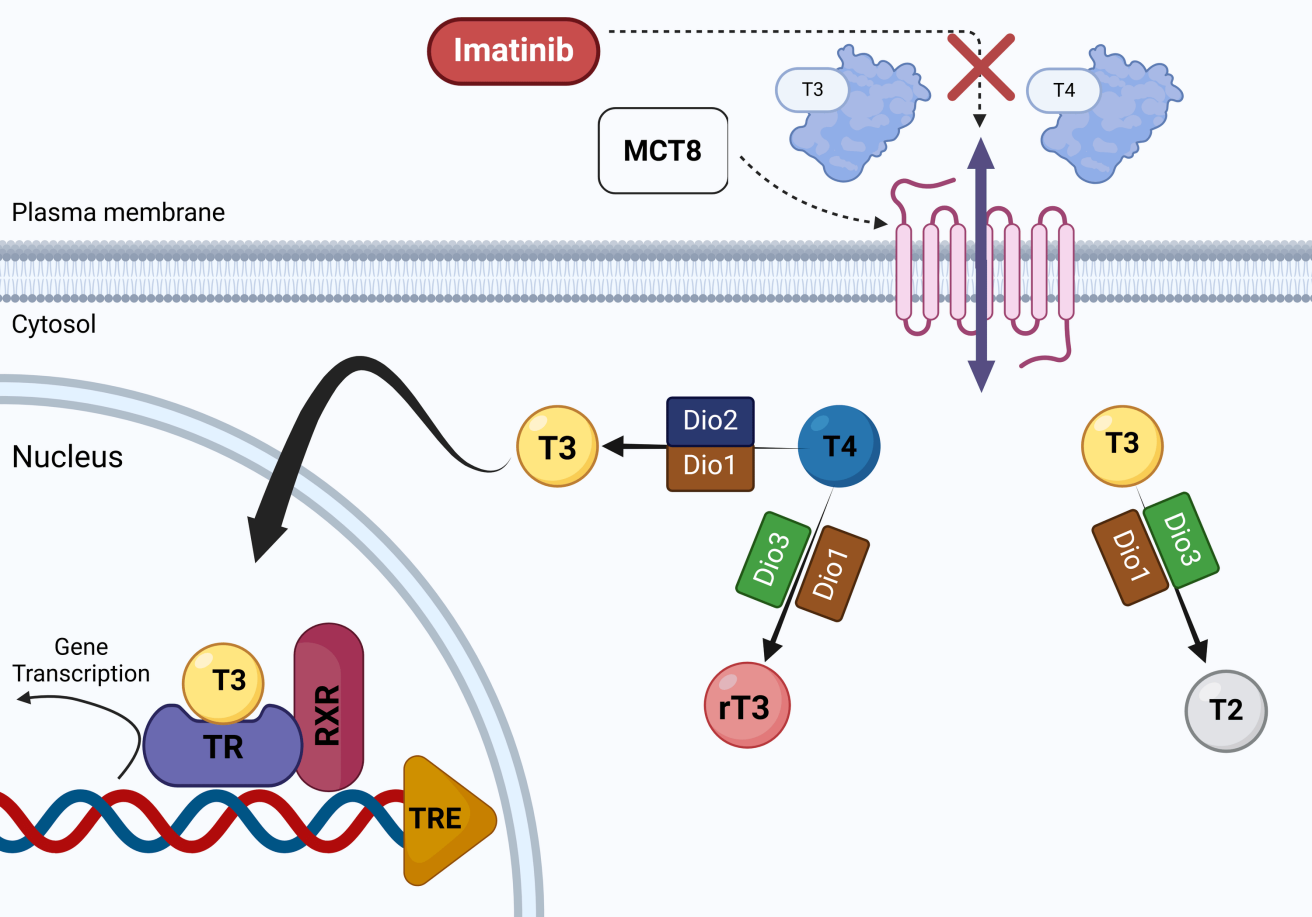
Illustration of how TKIs disrupt cellular uptake of T3 and T4 by inhibiting MCT8 Tyrosine kinase inhibitors (TKIs) have been shown to interfere with thyroid hormone transport and metabolism. Monocarboxylate transporter 8 (MCT8) enables the cellular uptake of thyroid hormones such as triiodothyronine (T3) and thyroxine (T4). Within cells, T4 can be converted into the active hormone T3 by type 1 deiodinase (Dio1) and type 2 deiodinase (Dio2) or into the inactive reverse T3 (rT3) by type 3 deiodinase (Dio3). T3 can also be metabolized to diiodothyronine (T2) by Dio1 or Dio3. Once inside the nucleus, T3 binds to the thyroid hormone receptor (TR), which forms a heterodimer with the retinoid X receptor (RXR). This complex interacts with the thyroid hormone response element (TRE) on DNA to regulate gene transcription. The figure is an original illustration by one of the authors. Image Credits: Nana Sardarova, MD

Multiple mechanisms have been suggested to explain TKI-related thyroid dysfunction, such as the induction of destructive thyroiditis, suppression of thyroid peroxidase (TPO) activity, altered absorption and metabolism of thyroid hormones, reduced iodide uptake, and regression of thyroid vasculature due to inhibition of VEGF [19-21]. Destructive thyroiditis is considered a primary mechanism and is often presented with an initial phase of hyperthyroidism, which is subsequently followed by hypothyroidism [22]. Commonly used TKIs in the treatment of CML, like imatinib and dasatinib, interfere noncompetitively with monocarboxylate transporter 8 (MCT8), a key protein responsible for the cellular uptake of thyroid hormones (Figure 3). This disruption impairs hormone entry into cells, resulting in abnormal thyroid function test results and elevated thyroid-stimulating hormone (TSH) levels [23]. Although limited data are available regarding the specific mechanisms by which other CML-associated TKIs affect thyroid function, several non-CML TKIs have been studied for their impact on thyroid pathophysiology. Therefore, these findings offer valuable insights and are considered relevant for inclusion in this discussion.

TKIs may upregulate the activity of type 3 deiodinase (Dio3), an enzyme that inactivates thyroid hormones by converting thyroxine (T4) into reverse triiodothyronine (rT3) and T3 into diiodothyronine (T2). This enhanced hormone breakdown results in decreased circulating levels of active T3 and T4 and a compensatory rise in TSH. This mechanism has been documented in studies involving sunitinib and sorafenib [24,25]. Another interesting effect was observed with vandetanib, which suppresses the activity of type 2 deiodinase (Dio2) in fibro/adipogenic progenitor cells, leading to decreased conversion of T4 to the biologically active hormone T3, which may contribute to the development of hypothyroidism [26]. Some other TKIs can cause regression of thyroidal capillaries by targeting vascular endothelial growth factor receptors (VEGFR), thus leading to hypothyroidism due to reduced thyroid blood flow [24]. Impaired iodine uptake by the thyroid gland was observed with other TKIs like sunitinib [27].

Thyroid Autoimmunity

Autoimmune thyroiditis is a condition in which the immune system targets the thyroid gland, causing infiltration by lymphocytes, progressive damage to thyroid tissue, and ultimately resulting in hypothyroidism. It is distinguished by the presence of thyroid-specific autoantibodies, specifically those directed against TPO antibodies (TPOAb) and thyroglobulin antibodies (TgAb) [28,29]. In the study by Rodia et al., 21 patients (30.4%) tested positive for TPOAb and/or TgAb (classified as ATA+) [9]. Among these, 10 individuals (47.6%) exhibited moderate hypoechogenicity on ultrasound, with one case of subclinical hypothyroidism. The other 11 ATA+ patients (52.4%) showed marked hypoechogenicity, with one experiencing subclinical hypothyroidism and two diagnosed with overt hypothyroidism [9]. Additionally, all 21 reported cases met the criteria for diagnosing Hashimoto's thyroiditis (HT). HT was found more often in patients using second-generation TKIs like nilotinib and dasatinib compared to those taking imatinib (43.75% vs. 18.9%; p = 0.03) [9].

Another case reported by Bakerywala et al. described a CML patient who developed destructive thyroiditis from nilotinib, which required discontinuation of the medication and treatment with steroids and beta-blockers [30]. In this case, initial laboratory workup showed low TSH, high total T3 and free T4, elevated thyroglobulin, strongly positive anti-thyroperoxidase antibodies, and a negative thyroid-stimulating immunoglobulin [30]. The impact of nilotinib on thyroid function was also evaluated by Kim and colleagues in a retrospective study, including 55 patients with Philadelphia chromosome-positive CML. Among the 30 patients who had no prior thyroid issues, four developed signs of thyroiditis, three of whom had positive anti-thyroid antibodies, characterized by a temporary phase of thyrotoxicosis followed by hypothyroidism [13].

Although the majority of reports described above are associated with nilotinib, we have identified two CML case reports of autoimmune thyroiditis due to imatinib. Singh et al. have reported a case of imatinib-induced autoimmune thyroiditis in a patient with CML who tested positive for thyroid microsomal antibodies (TMA) [31]. Another case of imatinib-induced autoimmunity was described by Wang et al. with acute onset of symptoms and markedly positive TPO and TgAb [32].

Thyroid Functions

Thyroid function is assessed through a combination of clinical evaluation, serological tests, and imaging studies. Measuring serum TSH and free T4 (FT4) levels is essential to distinguish types of thyroid dysfunctions.

Overt hyperthyroidism is characterized by low TSH levels and elevated FT4 or free T3 (FT3), as defined by laboratory-specific reference ranges, accompanied by clinical symptoms or the need for anti-thyroid treatment [14].

Subclinical hyperthyroidism refers to patients who are asymptomatic but present with suppressed TSH and normal FT4 and FT3 levels [14]. Overt hypothyroidism is indicated by an elevated TSH level and low FT4, along with clinical symptoms or the requirement for thyroid hormone replacement therapy [17]. Subclinical hypothyroidism is defined by a TSH level between 5 uIU/mL and 10 uIU/mL (or higher) with a normal FT4 concentration [6].

In the study conducted by Khaleel et al., thyroid function tests revealed that around 10% of patients on nilotinib had hypothyroidism, 3% had hyperthyroidism, and the remaining 87% had normal thyroid function [10]. However, none of the patients exhibited clinical symptoms of thyroid dysfunction [10]. Rodia and colleagues, in their cohort study, reported the occurrence of hypothyroidism in four patients (5.8%), with two cases of overt and two of subclinical hypothyroidism, all within the HT group [9]. Subclinical hyperthyroidism was observed in three patients (4.3%), mainly on imatinib, with one case likely due to TKI-induced destructive thyroiditis [9]. According to findings reported by Chawalitmongkol et al., 14.7% of CML patients showed abnormal thyroid function tests, with subclinical hypothyroidism being the most common pattern, most often linked to imatinib therapy [11]. Another study conducted in Nigeria by Ahmed et al. confirmed subclinical hypothyroidism in 14% of CML patients as an adverse effect of continuous imatinib use [12]. During the study of Kim et al., 45% of CML patients (33 out of 73) showed at least one abnormal thyroid function test, with 25% developing hypothyroidism and 29% developing hyperthyroidism [13]. Thyroid function test results remained consistent throughout the three-, six-, and 12-month follow-up periods [13]. Luna et al. reported that 85% of CML patients (14 individuals) in the study maintained a euthyroid state, while two of the 14 patients developed hyperthyroidism [14]. Walia et al. reported subclinical hypothyroidism in two of 20 patients (10%; 95% CI: 3-30%), with a mean imatinib treatment duration of 6.1 years [17]. Although most of the thyroid dysfunctions have been observed, particularly with imatinib and second-generation TKIs such as nilotinib, we have identified a case report by Price detailing hypothyroidism secondary to ponatinib therapy used in refractory CML [33].

Effect of Thyroid Alterations on Therapeutic Response in CML

Interest in the association between thyroid alterations and cancer response has grown following multiple studies that have demonstrated that TKI-induced hypothyroidism appears to be a prognostic marker for improved survival outcomes in patients with solid tumors. Particularly, a retrospective cohort study conducted by Lechner et al. [6] has highlighted the possible impact of TKI-induced thyroid abnormalities on progression-free survival (PFS) and overall survival (OS) in patients with solid tumors. Their findings showed that TKI-treated patients (including those receiving imatinib, nilotinib, or dasatinib) who developed overt hypothyroidism experienced improved OS [6].

The association between thyroid alterations and molecular response in CML patients on TKI treatment has been primarily described in the study by Rodia et al. [9]. The study found that patients with thyroid alterations had a significantly higher rate of deep molecular response (DMR) and a lower rate of major molecular response (MMR) compared to those without thyroid issues [9]. Specifically, HT was strongly associated with DMR, regardless of whether patients were euthyroid or hypothyroid (p = 0.0001 vs. MMR) [9]. This association persisted when analyzing patients on first- and second-generation TKIs separately. There was no link between DMR and non-autoimmune subclinical hyperthyroidism or thyroid nodules, the latter of which were mainly benign or indeterminate on fine-needle aspiration cytology (FNAC) [9].

The high incidence of thyroid autoimmunity observed in CML patients with more favorable outcomes may support the hypothesis that broad immune system activation could both promote the development of HT and enhance tumor control through a systemic immune response to cancer [9]. Supporting this idea, a study by Steegmann et al. [34] showed that dasatinib may exert an immunostimulating effect, contributing to a major molecular response in CML patients. Therefore, early identification of thyroid autoimmunity and autoimmune hypothyroidism could not only help detect cases of HT but also serve as a potential biomarker for the effectiveness of TKI therapy [9].

Clinical Approach to Managing Thyroid Dysfunction

While it is well-established that TKIs can lead to thyroid dysfunction and may even have prognostic significance, there are currently no guidelines outlining how frequently thyroid function should be monitored or how to manage TKI-induced thyroid abnormalities [5]. The National Comprehensive Cancer Network (NCCN) recommends evaluating thyroid function by checking TSH and FT4 levels before starting TKI therapy [35]. During the first six months of treatment, thyroid function should be closely monitored with TSH and FT4 tests every four to six weeks, as alterations in FT4 may occur earlier than changes in TSH [36,37].

Regarding the threshold for initiating treatment based on elevated TSH levels, the American Thyroid Association, the American Association of Clinical Endocrinologists, and the Endocrine Society advise starting thyroid hormone therapy in the presence of hypothyroid symptoms and/or cardiovascular issues, positive TPO antibodies, or when TSH exceeds 10 uIU/mL [12]. Additional indications for starting treatment include being aged 60 or older and during pregnancy [29]. However, there is limited evidence to guide treatment decisions for TSH levels between 4.5 and 10 uIU/mL [5]. In most of the studies included in this review, patients primarily exhibited subclinical hypothyroidism without any accompanying symptoms and, therefore, did not require treatment. Concerning the decision of whether TKI therapy should be continued or discontinued, Chawalitmongkol et al. reported that the initial TKI dose was maintained in all patients except for those who experienced disease progression [11]. Neither the number nor the duration of TKI treatments showed a significant correlation with abnormalities in thyroid function tests [11]. Clinically significant symptoms were also rare in the study by Kim et al., and most patients continued TKI therapy without needing thyroid-specific treatment [13]. Three patients required treatment: two hypothyroid patients started levothyroxine, one of whom was on amiodarone with slightly elevated baseline TSH, and one patient on nilotinib briefly received methimazole before developing transient hypothyroidism [13]. TKI therapy was discontinued in 93% of patients with overt hypothyroidism in the study by Luna et al. [14]. All patients diagnosed with overt hypothyroidism received thyroid hormone treatment (p = 0.023) and were referred to an endocrinology specialist (p = 0.002) [14]. TKI therapy was restarted once biochemical euthyroidism was achieved [14]. Among patients with transient hypothyroidism, 88% recovered spontaneously without thyroid hormone replacement, with an average recovery time of 40 months [14].

As mentioned previously, there are currently no established guidelines for managing TKI-induced thyroid abnormalities in patients with CML, highlighting the need for further research. Interestingly, Garfield et al. advised caution regarding the routine administration of thyroid hormone replacement, noting it could potentially promote tumor growth in patients with active cancers, particularly given evidence suggesting that hypothyroidism might have a protective effect in these patients [38]. While Garfield et al. recommend against routine hormone replacement therapy in CML patients [38], this approach may not be universally applicable, as the decision remains context-dependent and is still debated within the field.

Limitations

The review was limited by the relatively small number of eligible studies after applying the inclusion and exclusion criteria. Most included studies were observational, potentially introducing selection bias or confounding factors that may affect the generalizability of the conclusions. The lack of randomized controlled trials restricts the strength of the evidence and limits definitive conclusions regarding management strategies and recommendations. It’s important to note that this review was conducted as a systematic review without meta-analysis due to the considerable variability in study designs, patient populations, and outcome definitions among the included studies. These differences limited the feasibility and validity of statistical pooling or meta-regression. In accordance with PRISMA 2020, a narrative synthesis was employed. While this limits the ability to produce summary effect estimates, it allows for a structured interpretation of available evidence. Additionally, it is important to acknowledge the potential for publication bias, particularly the underreporting of negative or inconclusive findings in smaller observational studies, which may have influenced the overall interpretation of the evidence. Future research should focus on prospective, multicenter studies with standardized thyroid monitoring protocols and transparent reporting of both positive and negative findings to strengthen the quality and applicability of the evidence.

Although our data search was conducted through November 30, 2024, we acknowledge that several relevant studies have been published since that time. These newer data offer important insights into emerging agents and may further refine prevalence estimates and monitoring strategies. However, to maintain methodological consistency and transparency, these studies were not included in the present review. Future systematic reviews or meta-analyses may consider integrating these newer findings to update and expand the current evidence base.

## Conclusions

In conclusion, this systematic review highlights that thyroid dysfunction, including both hypothyroidism and hyperthyroidism, is a frequent complication among patients with CML treated with TKIs. Most cases reported were subclinical, transient, and rarely required specific therapeutic intervention. However, overt hypothyroidism consistently necessitated thyroid hormone replacement therapy and endocrine referral. The relationship between thyroid alterations, particularly autoimmune thyroiditis, and improved molecular responses suggests that these thyroid-related side effects could serve as biomarkers of therapeutic efficacy in CML patients undergoing TKI treatment. Incorporating routine thyroid function monitoring into clinical management pathways and clinical trial protocols for CML patients may facilitate early detection of thyroid dysfunction and promote standardized, proactive care.

Despite these insights, the review underscores notable limitations, such as the predominance of observational study designs, variability in patient characteristics, differences in definitions and management protocols for thyroid dysfunction, and the absence of randomized controlled trials. There are currently no universally accepted guidelines for monitoring or treating thyroid abnormalities induced by TKIs in CML patients, emphasizing the need for additional prospective research to establish evidence-based clinical guidelines. Such guidelines would enable clinicians to effectively balance the benefits of continuing TKI therapy against the potential risks associated with thyroid dysfunction.
